# Fed-Batch Strategy Achieves the Production of High Concentration Fermentable Sugar Solution and Cellulosic Ethanol from Pretreated Corn Stover and Corn Cob

**DOI:** 10.3390/ijms252212306

**Published:** 2024-11-16

**Authors:** Jiamin Huang, Xuezhi Li, Jian Zhao, Yinbo Qu

**Affiliations:** State Key Laboratory of Microbial Technology, Shandong University, No. 72, Binhai Road, Qingdao 266237, China; jiaminhuang_220@163.com (J.H.); lixz@sdu.edu.cn (X.L.); quyinbo@sdu.edu.cn (Y.Q.)

**Keywords:** enzymatic hydrolysis, high solid loading, fed-batch, lignocellulosic biomass, fermentable sugar, ethanol

## Abstract

The bioconversion of lignocellulosic biomass, which are abundant and renewable resources, into liquid fuels and bulk chemicals is a promising solution to the current challenges of resource scarcity, energy crisis, and carbon emissions. Considering the separation of some end-products, it is necessary to firstly obtain a high concentration separated fermentable sugar solution, and then conduct fermentation. For this purpose, in this study, using acid catalyzed steam explosion pretreated corn stover (ACSE-CS) and corn cob residues (CCR) as cellulosic substrate, respectively, the batch feeding strategies and enzymatic hydrolysis conditions were investigated to achieve the efficient enzymatic hydrolysis at high solid loading. It was shown that the fermentable sugar solutions of 161.2 g/L and 205 g/L were obtained, respectively, by fed-batch enzymatic hydrolysis of ACSE-CS under 30% of final solid loading with 10 FPU/g DM of crude cellulase, and of CCR at 27% of final solid loading with 8 FPU/g DM of crude cellulase, which have the potential to be directly applied to the large-scale fermentation process without the need for concentration, and the conversion of glucan in ACSE-CS and CCR reached 80.9% and 87.6%, respectively, at 72 h of enzymatic hydrolysis. This study also applied the fed-batch simultaneous saccharification and co-fermentation process to effectively convert the two cellulosic substrates into ethanol, and the ethanol concentrations in fermentation broth reached 46.1 g/L and 72.8 g/L for ACSE-CS and CCR, respectively, at 144 h of fermentation. This study provides a valuable reference for the establishment of “sugar platform” based on lignocellulosic biomass and the production of cellulosic ethanol.

## 1. Introduction

Globally, with the escalating demand for resources and energy in human society, fossil resources face the risk of depletion. Lignocellulosic biomass such as grasses and agricultural waste offer the advantages of an abundant and widely available supply as well as an infinitely renewable potential, despite being currently underutilized [1]. There has been an increasing interest in the production of liquid fuels such as second-generation bioethanol and bulk chemicals from lignocellulosic biomass through bioconversion process [2,3,4,5]. Generally, the bioconversion of lignocellulose included three main steps, pretreatment, enzymatic hydrolysis, and fermentation. Of those steps, using enzymes (cellulase and hemicellulases) to hydrolyze lignocellulose for producing fermentable sugars is a key step. Considering the separation of some end-products, it is necessary to obtain a high concentration separated sugar solution instead of a solid-liquid mixture obtained by enzymatic hydrolysis without separation to use in subsequent fermentation process, that is to say, establishing the “sugar platform” based on lignocellulosic biomass [6]. To achieve it, enzymatic hydrolysis must be conducted at a high solid loading. Meanwhile, this process can also offer other several advantages, including using fewer number of reactors or smaller equipment for producing the same output, decreasing energy and water consumption, and reducing the investment and production costs associated with large-scale production [7,8]. However, as the solid loading increases, some insignificant issues in the enzymatic hydrolysis at low solid loading become more significant [9]. For example, during enzymatic hydrolysis of lignocellulose, water is crucial since it mediates enzymes and products diffusion from reaction sites and glycosidic bonds [10], increasing the hydrolysis efficiency. When enzymatic hydrolysis was conducted at high solid loading, available water declined in the reaction system, and the apparent viscosity of substrates rises, making it difficult for the fibrous substrates and enzymes to mix evenly, affecting the efficiency of enzyme action. Moreover, the hydrolysis at high-loading resulted in the accumulation of products such as cellobiose and glucose, which can inhibit the activities of cellobiose hydrolase, endoglucanase, and β-glucosidase [11]. With the lignocellulosic substrate concentration increases, this phenomenon becomes more pronounced.

To overcome these shortcomings, fed-batch process is usually used in the enzymatic hydrolysis at high solid loading. In this process, the enzymatic hydrolysis is firstly conducted at a low substrate content; subsequently, cellulosic substrate and/or enzyme are added to the hydrolysis system in batches for maintaining a low slurry viscosity in the system while achieving the desired final substrate concentration during enzymatic hydrolysis. This batch feeding method does not require changing the original reactor but addresses the mass transfer issue resulting from high viscosity at high solid loadings by improving the operation mode. Moreover, it helps maintain a stable water content in the reaction system and promotes thorough mixing of enzyme and substrate [12]. Compared to the enzymatic hydrolysis where cellulosic substrates and enzymes are added all at once at the beginning of the reaction, this batch feeding enzymatic hydrolysis method can enhance conversion efficiency of lignocellulose to some extent [13]. For example, Kim et al. used fed-batch enzymatic hydrolysis process to hydrolyze pretreated oak, in which the initial solid loading was 15% (*w*/*w*), and the Cellic CTec2 enzyme of 30 FPU/g glucan was added all at once to the slurry at 0 h, feeding the cellulosic substrate to the system twice, at 12 h and 24 h, respectively, until the final solid loading reached 30% (*w*/*w*), and the final saccharification yields at 120 h reached 75.9% and 58.6% of the theoretical maximum yields of glucose and xylose, respectively [14]. Gong et al. used alkaline organosolv-pretreated corn stover as substrate, along with the enzyme blend containing cellulase, β-glucosidase, and hemicellulase of 15 mg protein/g glucan; enzymatic hydrolysis was started at 12% (*w*/*v*) of solid loading, and 7% of fresh substrate was fed at 3, 6, 24, and 48 h, respectively, to achieve a final solid loading of 40% (*w*/*v*). During feeding, the enzyme mixture was also fed into the system simultaneously with the pretreated corn stover in the corresponding proportion. Finally, the conversion of glucan and xylan reached 89.5% and 88.5% at 120 h, respectively, when the oligosaccharides were taken into account [15]. Xu et al. used alkaline-pretreated sugarcane bagasse as substrate, by optimizing a fed-batch strategy that initially used a 10% (*w*/*v*) substrate dosage and then added 5%, 4% and 3% (*w*/*v*) at 8, 12 and 16 h, respectively. As a result, 122 g/L of glucose titer and 80% of glucose yield were achieved at 22% (*w*/*v*) solid loading after enzymatic hydrolysis of 48 h [16]. Although the fed-batch enzymatic hydrolysis has been investigated, there are still issues such as high total enzyme dosages, low total sugar content in hydrolysates, etc. To construct the sugar platform used for bioconversion of lignocellulose, it is crucial to produce the high concentration fermentable sugar solution under a low enzyme dosage, which is beneficial for the separation of subsequent fermentation products and a reduction in the total cost of bioconversion. It was reported that, when utilizing the batch feeding method, the feeding strategy and feeding time can significantly impact the ultimate efficacy of enzymatic hydrolysis [17]; therefore, targeted optimization must be carried out for specific lignocellulosic substrates.

The simultaneous saccharification and co-fermentation (SSCF) together form a promising process for the production of cellulosic ethanol using lignocellulosic biomass as feedstock. In this process, enzymatic hydrolysis and co-fermentation of glucose and xylose can occur simultaneously, eliminating the inhibitory effect of the products glucose and xylose on enzymatic hydrolysis, while reducing total processing time, and minimizing contamination risks [18,19]. Considering energy consumption and ethanol recovery efficiency, a concentration of 4% (*w*/*v*) ethanol in the fermentation broth serves as an efficient distillation benchmark [20]. This requires SSCF to be performed at a higher cellulosic substrate concentration. Nevertheless, as solid loading increases in SSCF, ethanol production declines due to heightened mass transfer resistance and reduced xylose absorption [21,22]. Applying a fed-batch mode during running SSCF can preserve a low viscosity in the reaction system, enabling efficient mixing of enzymes and substrates, thereby leading to elevated ethanol concentrations. Furthermore, the implementation of batch feeding for SSCF harbors the potential to sustain a low glucose/xylose ratio, which proves advantageous in facilitating xylose uptake [23].

Corn stover is one of the most abundant agricultural wastes, which is ubiquitous all over the world. It is an important lignocellulosic material with the advantages of renewable, large output, wide distribution and low price [24,25]. Steam explosion pretreatment has significant advantages in energy consumption, pretreatment effect, and environmental friendliness, making it one of the pretreatment methods with promising application prospects in industry [26,27]. The pretreatment of corn stover using steam explosion can better disrupt the network structure between lignocellulose and increase enzyme accessibility [28]. Wang et al. used glycerol-assisted steam explosion to pretreat corn stover [29] and showed that the glucose yield increased from 54.8% to 79.3% under identical enzymatic hydrolysis conditions. Chen et al. used steam explosion to pretreat corn stover under optimized pretreatment conditions, and the glucose yield increased by about 55% compared with untreated corn stover [30]. Corncob has become an important feedstock for industrial production of xylose, xylo-oligosaccharides, xylitol, and other products, but a large number of corncob residues (CCR) were produced in the production process [31,32]. In China, over five million tons of CCR were generated annually, most of which were usually burned and cannot be fully utilized [33]. Due to the effective removal and utilization of hemicellulose, the cellulose content in CCR was higher (about 55–75%) [34,35], and its structure was looser than that of natural corn cob, making it easier to be hydrolyzed by enzyme [36]. Using CCR to produce fermentable sugar can not only reduce the “carbon emissions” caused by direct incineration of CCR, but it also has important significance for promoting local economic development.

In this article, using acid catalyzed steam explosion pretreated corn stover (ACSE-CS) and corn cob residues obtained through extracting xylose from corn cob with dilute sulfuric acid, respectively, as feedstocks, the fed-batch enzymatic hydrolysis of the substrates were optimized to produce high concentration fermentable sugar liquid, mainly, focused on the impact of different feeding strategies. Additionally, using a domesticated *Saccharomyces cerevisiae* LFB-1, which has the ability to co-metabolize glucose and xylose, the fed-batch SSCF of the feedstocks for producing cellulosic ethanol was also investigated. This study will help further promote the resource utilization of corn stover and corn cob residues by bioconversion, and it provides a valuable reference for the establishment of “sugar platform” based on lignocellulosic biomass and the production of cellulosic ethanol.

## 2. Results and Discussion

### 2.1. Chemical Compositions of Lignocellulosic Substrates

The chemical compositions of two cellulosic substates used in this study, ACSE-CS and CCR, were firstly determined, and the results are shown in Table 1.

It was shown that the ACSE-CS contains 37.8% of glucan, 18.6% of xylan, and 14.7% of lignin. In addition, it also contains about 12.6% of ash and about 17.3 of extractives by ethanol. Soluble xylo-oligosaccharides of about 13.15% were detected by HPLC in the extraction solution after extracting the pretreated corn stover with water, which should be attributed to acidic degradation of xylan in corn stover during pretreatment. The literature has also been reported that the hemicellulose can be degraded into oligosaccharides and monosaccharides during steam explosion pretreatment, and even some xylose was further dehydrated to form furfural, a toxic substance that inhibits microorganisms used in subsequent fermentation [37,38]. Under acidic environment with low pH, the degradation of hemicellulose will be more severe, resulting in more soluble hemicellulose sugar production. The CCR was obtained after pretreatment of corn cob with dilute acid and ammonium sulfite, and during this pretreatment process, a significant amount of xylan and lignin was degraded and removed, resulting in a high cellulose content of 75.1% in the CCR, and relative low lignin content of 8.67%. The two-step pretreatment can allow the cellulose in the corn cob to be more exposed, and meanwhile, a low content lignin can decrease the non-productive absorption of lignin to enzyme, which was considered one of the key reasons affecting the efficiency of enzymatic hydrolysis of lignocellulose [39,40,41]. These features of CCR suggested that it may be easily hydrolyzed by cellulases.

### 2.2. Fed-Batch Enzymatic Hydrolysis of Cellulosic Substates to Produce High Concentration Fermentable Sugar Solution

To obtain a high concentration fermentable sugar solution, the enzymatic saccharification must be conducted at high solid loading. It has been proved that employing batch feeding techniques was an effective approach to enhance the enzymatic hydrolysis efficiency of non-wood lignocellulosic materials at high solid loadings [42]. Due to the difference in characteristics of various lignocellulosic materials, it was necessary to optimize the respective feeding mode and enzymatic hydrolysis conditions for different cellulosic substrates.

#### 2.2.1. Fed-Batch Enzymatic Hydrolysis of ACSE-CS

##### Effect of Batch Feeding Strategy

**Figure 1:**
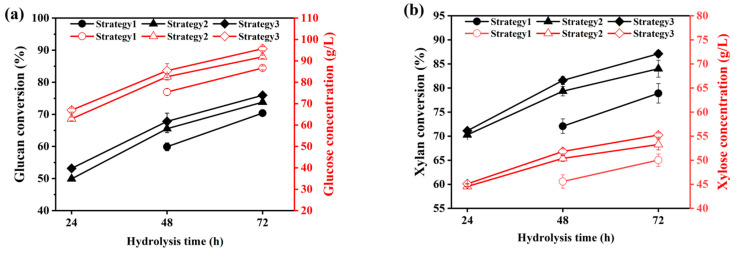
Effect of various fed-batch strategies on the glucan conversion and glucose concentration (**a**), and the xylan conversion and xylose concentration (**b**) during enzymatic hydrolysis of ACSE-CS. Note: the solid symbols represent the conversion rate, and the hollow symbols represent the sugar concentration.

The batch feeding strategy influenced the final enzymatic hydrolysis efficiency [43]. In this study, three different batch feeding strategies were employed, as shown in Table 2, to reach the final solid loading of 30% in hydrolysis system. For the first feeding mode, the initial solid loading was set at 10%, with feeding occurring at a rate of 7% every 8 h, and after three rounds of feeding, the final solid loading reached 30%. In this process, cellulase was added in batches according to a certain proportion of substrate weight (g, DM)/enzymatic activity (FPU). In the second and the third feeding modes, the initial solid loading was increased to 15%, and then to 23% and 30% through two rounds of feeding at 3 h and 6 h of enzymatic hydrolysis, respectively, which was based on the observation that the initial substrate in system can be completely liquefied after 3 h of hydrolysis. The difference between the second and third feeding mode was that cellulase was added in batches according to a certain proportion in the second mode, while in the third mode, cellulase was added all at once to the reaction system. Figure 1 illustrated the enzymatic hydrolysis effect of ACSE-CS by using various feeding strategies.

Figure 1 shows that, whether it is glucan or xylan, using the third feeding mode obtained the highest conversion, specifically 76.1% for glucan and 87.1% for xylan, and for the maximum sugar yields, the concentrations of glucose and xylose in hydrolysis liquid reached 95.9 g/L and 55.2 g/L, respectively. The first feeding mode resulted in the worst enzymatic hydrolysis effect, and meanwhile, poor material liquefaction was observed during the initial 48 h of the enzymatic hydrolysis. The possible reasons for this phenomenon were that (1) the insufficient initial addition of enzyme in the first feeding mode cannot cause rapid degradation of cellulose into short-chain oligosaccharides, but in the third feeding mode, as the cellulase was added all at once at the beginning stage of enzymatic hydrolysis, the excessive cellulase action resulted in a rapid decrease in the polymerization degree of cellulose, thus forming a liquefied state; and (2) too long of a feeding interval potentially resulted in enzyme inhibition due to the formation of inhibitory products such as cellobiose, xylo-oligosaccharides, and so on [44,45,46]. This inhibitory effect may persist until the addition of more substrate and enzyme [47]. Xu et al. reported that, during high-solid-loading enzymatic hydrolysis of alkali-pretreated sugarcane bagasse by fed-batch strategy, feeding all biomass within 20–22 h may be beneficial for preventing the decrease in enzyme activity over time [16]. Shiva et al. also found that using short time intervals for feeding improved the efficiency of enzymatic hydrolysis of biomass under high solid loading [7]. The results indicated that, when using the batch feeding method to conduct enzymatic hydrolysis of lignocellulose at high solid loading, adding sufficient enzyme to the reaction system in the initial stage of enzymatic hydrolysis should be beneficial, as it can promote rapid liquefaction of solid substrate while eliminate inhibition of process products, thus achieving high enzymatic hydrolysis efficiency. And the feeding operations may be conducted according to the liquefaction status.

##### Effect of Tween 80 Addition on Enzymatic Hydrolysis of ACSE-CS

**Figure 2:**
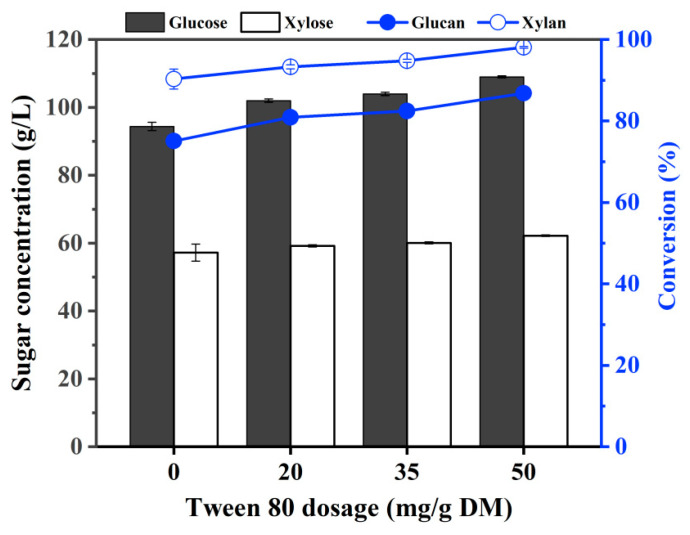
Effect of Tween 80 additions on the concentrations of glucose and xylose in hydrolysis system, and conversions of glucan and xylan at 72 h of enzymatic hydrolysis of ACSE-CS. Here, the columns represent sugar concentrations, and the lines represent conversion rates. Note: The enzymatic hydrolysis was carried out under the conditions of 30% (*w*/*v*) of solid loading, total cellulase dosage of 10 FPU/g DM, 48 °C, pH 4.8, 150 rpm in a reaction system of 20 mL in 50 mL flask.

Previous researches had demonstrated the positive impact of adding Tween 80 as a surfactant on the enzymatic conversion efficiency of pretreated corn stover by reducing non-productive adsorption of enzyme onto lignin [48,49]. It was reported that Tween 80 can change the physical properties of lignin and increase its hydrophilicity, thereby forming a hydrated layer on the surface of lignin as a steric hindrance to the non-productive adsorption of cellulase onto lignin [50]. Due to the high lignin content in the ACSE-CS (Table 1), in this study, we tried to further improve enzymatic hydrolysis of ACSE-CS by adding Tween 80. The selected additions of Tween 80 were 20 mg/g DM, 35 mg/g DM, and 50 mg/g DM, respectively, and its effect on enzymatic hydrolysis are presented in Figure 2.

Figure 2 demonstrated the improvement in enzymatic hydrolysis efficiency by the addition of Tween 80. With the increase in the additions of Tween 80, the concentrations of produced fermentable sugars and the conversions of glucan and xylan increase. By adding 20 mg/g DM of Tween 80, the glucan conversion reached 80.9% at 72 h of enzymatic hydrolysis, and the total fermentable sugar concentration in hydrolysis liquid reached 161.2 g/L, in which the concentrations of glucose and xylose were 102 g/L and 59.2 g/L, respectively. This hydrolysis liquid with the sugar concentration met the requirements for subsequent process, and it can be directly used to ferment for producing final products such as ethanol without the need for concentration. Zhang et al. added 150 mg/g substrate of Tween 80 to the NaOH-pretreated sugarcane bagasse enzymatic hydrolysis system, and the glucose yield at 24 h increased by 5.1% [51]. Huang et al. found the enzymatic digestibility of pretreated bamboo was increased significantly from 29.4% to 61.6% when 0.3 g/g of Tween 80 was supplemented during the enzymatic hydrolysis [50]. However, in their study, the addition of Tween 80 was too high to be applied in large-scale production. In this research, adding a small amount of Tween 80 (20 mg/g DM) achieved the efficient conversion of ACES-CS with high lignin content, resulting in the production of fermentable sugars with high sugar concentrations (Total sugar exceeds 160 g/L by direct enzymatic hydrolysis).

#### 2.2.2. Fed-Batch Enzymatic Hydrolysis of CCR

**Figure 3:**
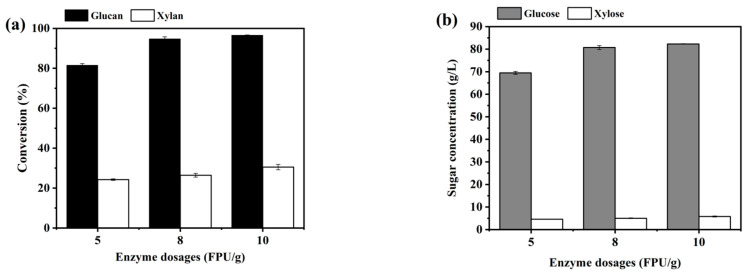
The effect of cellulase dosages on the conversions of glucan and xylan (**a**) and the concentrations of glucose and xylose in hydrolysis system (**b**) at 72 h of enzymatic hydrolysis of CCR.

The influence of cellulase additions on enzymatic hydrolysis of CCR was firstly investigated. Three enzyme dosages, namely 5 FPU/g DM, 8 FPU/g DM, and 10 FPU/g DM, respectively, were selected and the enzymatic hydrolysis experiments were carried out under 10% (*w*/*v*) of solid loading. The results are presented in Figure 3. It was shown that, using 8 FPU/g DM of enzyme, the contents of glucose and xylose in hydrolysis system were 80.7 g/L and 5.7 g/L, respectively, after 72 h of enzymatic hydrolysis, and at this time, the glucan conversion reached 94.7%. Further increasing the enzyme dosage to 10 FPU/g DM did not significantly improve the glucan conversion, which was due to the near-complete degradation of glucan in the substrate.

Dai et al. investigated the enzymatic hydrolysis of active oxygen and solid alkali/dilute sulfuric acid-pretreated corn cob at different enzyme dosages (5, 10, 15, 20, 25 FPU/g of substrate), and found that the conversion of cellulose reached 98.88%, and glucose content in hydrolysis liquid was 36.47 g/L when enzymatic hydrolysis was conducted at 5% of solid loading for 72 h with 25 FPU/g of enzyme [52]. Li et al. conducted enzymatic saccharification of dilute-acid-pretreated-corn cobs, and found that, under the solid loading of 10% (*w*/*v*) and cellulase dosage of 15 FPU/g solid, the glucose yield was 94.2% and the glucose concentration was 53.7 g/L after 72 h of enzymatic hydrolysis [53]. Compared to these results, in this study, a higher sugar concentration was obtained by using a lower cellulase dosage (8 FPU/g).

Further, fed-batch enzymatic hydrolysis of the CCR was conducted to obtain fermentable sugar solution for bioconversion. Based on the results of pre-experiments, the initial solid loading was set at 15% (*w*/*v*), and the required amount of cellulase (8 FPU/g DM) was added all at once to the reaction system at the beginning of enzymatic hydrolysis. The feeding operations were conducted at 4 h and 7 h of enzymatic hydrolysis, respectively, which were based on the observation of the liquefaction state of the cellulosic substrates. After feeding substrate, the solid loading in reaction system reached to 21% (*w*/*v*) and 27% (*w*/*v*), respectively. A total of 20 mg/g DM of Tween 80 was also added into this enzymatic hydrolysis system at initial stage.

**Figure 4:**
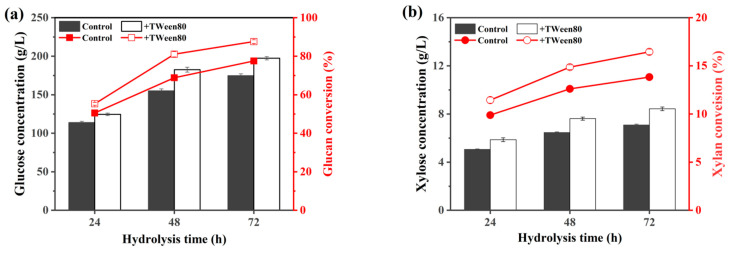
Changes of glucan conversion and glucose concentration (**a**), and xylan conversion and xylose concentration (**b**) during fed-batch enzymatic hydrolysis of CCR at a final solid loading of 27%, in which the additions of Tween 80 and enzyme were 20 mg/g DM and 8 FPU/g DM, respectively. Note: the histogram represents the concentration, and the line represents the conversion.

The results of enzymatic hydrolysis, as shown in Figure 4, indicated that the addition of Tween 80 improved the enzymatic hydrolysis of CCR, and the conversions of glucan and xylan at 72 h of enzymatic hydrolysis increased from 77.6% to 87.6% and from 13.8% to 16.5%, respectively. The final glucose concentration in hydrolysis liquid reached 197 g/L, and the total sugar concentration amounted to 205 g/L at solid loading of 27% (*w*/*v*). Zanuso et al. used fed-batch enzymatic hydrolysis process to hydrolyze hydrothermal-pretreated corn cobs. The initial solid loading was set at 8.3%, with 8.3% cellulosic substrate being fed every 6 h, and after two rounds of feeding, the final solid content reached 25%. The enzyme loading was 10 FPU/g substrate, and total enzyme was added at once at the beginning of the reaction. After a total of 48 h of enzymatic hydrolysis, 170 g/L of glucose was obtained [54]. Cai et al. adopted a “low amount and high frequency” batch feeding mode: 10%-3%-3%-3%-3%-3%, that is, the initial solid loading was set at 10%, with 3% cellulosic substrate being fed every 5 h, and after five rounds of feeding, the final solid loading reached 25%. This mode was confirmed to be optimal for enzymatic hydrolysis system of acidic pretreated corncobs with a final solid loading of 25% under a cellulase addition of 7.3 FPU/g dry substrate, resulting in a glucose yield of 84.4% at 96 h of enzymatic hydrolysis [55]. Compared these results, a higher concentration of sugar solution and a higher conversion rate were obtained by the fed-batch strategy used in this research.

### 2.3. Fed-Batch SSCF of ACSE-CS and CCR for Ethanol Production

#### 2.3.1. Selection of Fermentation Temperature

**Figure 5:**
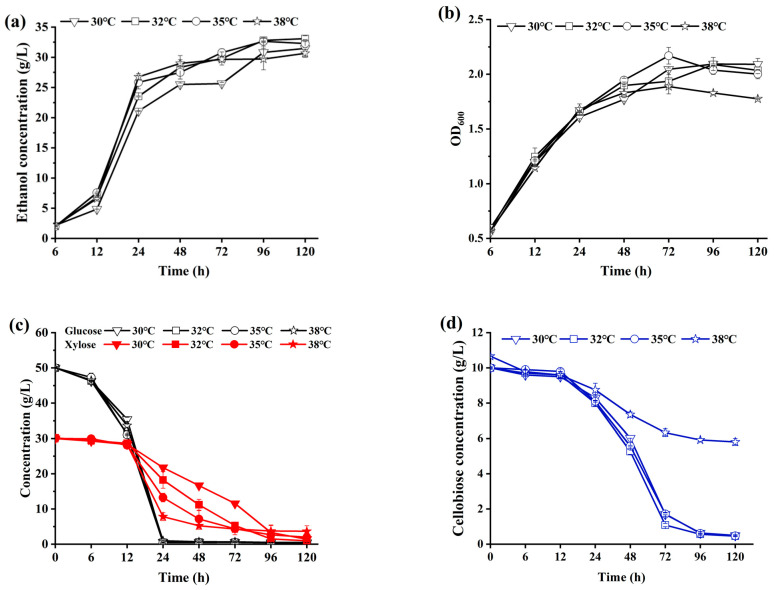
The effect of culture temperature on ethanol concentration (**a**), OD600 (**b**), glucose and xylose concentrations (**c**), and cellobiose concentration (**d**) in the fermentation broths during fermentation of A. LFB-1 using prepared pure sugar medium.

The domesticated *Saccharomyces cerevisiae* LFB-1, which has an ability to co-metabolize glucose and xylose, was used in this study for producing ethanol through the fed-batch SSCF process without detoxification. Initially, an investigation was conducted to determine the optimal fermentation temperature for the strain A. LFB-1 using the prepared pure sugar culture medium containing glucose of 50 g/L, xylose of 30 g/L, and cellobiose of 10 g/L, which was based on the concentrations of the sugars obtained from the enzymatic hydrolysis of ACSE-CS at 30% of solid loading. The variations in the ethanol concentration and OD_600_ value over the fermentation time under the different fermentation temperatures were depicted in Figure 5. Additionally, the concentrations of residual sugars in the fermentation broths were also determined and are shown in Figure 5.

Figure 5 showed that the highest ethanol concentration was obtained at 35 °C, and at this time, the low residual sugar concentrations in fermentation system were detected, indicating the strain A. LFB-1 exhibited efficient sugar metabolism under this temperature. Consequently, 35 °C was selected as the fermentation temperature for subsequent SSCF experiment.

#### 2.3.2. Fed-Batch SSCF of ACSE-CS for Producing Ethanol

**Figure 6:**
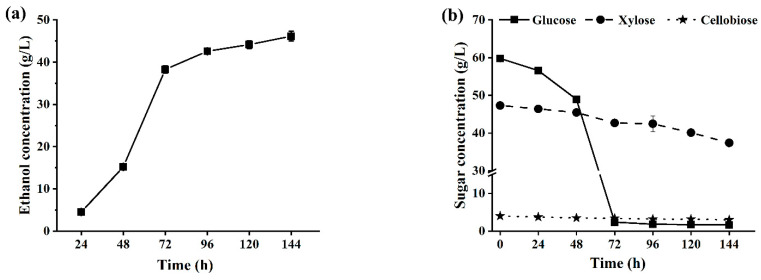
Changes of the ethanol concentration (**a**) and residual sugar concentrations (**b**) in fermentation broths during fed-batch SSCF of ACSE-CS. Note: the cellulase of total 10 FPU/g DM was used.

Initially, the solid concentration was set at 15%, and pre-saccharification of 3 h was conducted at 48 °C. Then, the system temperature was reduced to 35 °C for conducting SSCF by inoculating *Saccharomyces cerevisiae* A. LFB-1. The first and second feeding were carried out at 3 h and 6 h of fermentation, respectively, and after feeding, the solid loading in the fermentation system increased to 25%, and 35% respectively. Figure 6 showed the changes on the concentrations of ethanol and residual sugars in fermentation broths during SSCF.

Figure 6a showed that the ethanol concentration exhibited a swift rise within 24–72 h, and then slowly increased. This was consistent with the changes in the residual sugar concentrations in the fermentation broths (Figure 6b). It was observed that the glucose was rapidly consumed before 72 h of fermentation, and the concentration of xylose and cellobiose exhibited a gradual decrease throughout the entire fermentation period. During the initial 48 h of fermentation, the utilization rate of glucose was relatively slower than that after 48 h, which may be attributed to the strain’s metabolic capacity being affected by the high concentration of inhibitors at elevated solid concentration. At 144 h of fermentation, the concentration of ethanol in the fermentation broth reached 46.1 g/L. In a report from Zhu et al., using dry diluted acid-pretreated corn stover as a substrate achieved an ethanol concentration of 47.2 g/L at 25% (*w*/*v*) solid content through fed-batch SSCF without detoxification, but with increased yeast inoculation [56]. On the other hand, in this experiment, at the end of fermentation, it was found that the fermentation broth still contained a certain amount of cellobiose and xylose. In subsequent studies, by improving cellulase system components, for example, increasing β-glucosidase ratio in cellulase complex to degrade cellobiose to glucose, and further enhancing the xylose metabolism ability of the yeast strain A. LFB-1, it is expected that the ethanol concentration will increase further.

#### 2.3.3. Fed-Batch SSCF of CCR for Producing Ethanol

In the fed-batch SSCF of CCR, the same process operation identical to SSCF of ACSE-CS was completed, but the first and the second feeding were conducted at 4 h and 9 h of fermentation, respectively, which were determined based on the completion of substrate liquefaction, and the solid loadings in reaction system reached 23% and 29%, respectively, after two rounds of feeding.

**Figure 7:**
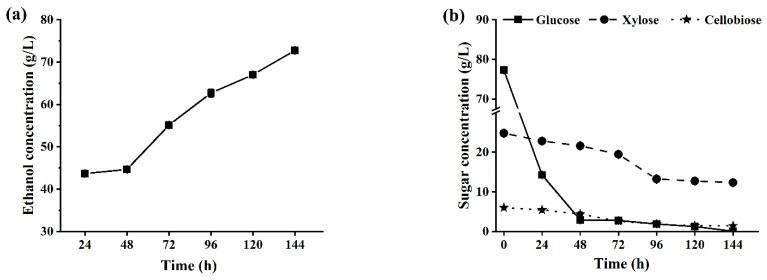
Changes of ethanol concentration (**a**), and residual sugar concentrations (**b**) in fermentation broths during fed-batch SSCF of CCR. Note: total cellulase dosage was 8 FPU/g DM, and the solid loading was 29% (*w*/*v*).

Figure 7 showed that, during the SSCF of CCR, similar changes in the concentrations of ethanol and residual sugars in fermentation broths were observed as in the SSCF of ACSE-CS, but a higher ethanol concentration of 72.8 g/L in the fermentation broth was obtained at 144 h of fermentation, indicating that CCR was a better raw material than ACSE-CS for cellulosic ethanol production. Hai et al. performed the SSCF of alkali-pretreated corn cob at 20% (*w*/*v*) solid content, and ethanol concentration was 53.24 g/L at 84 h of fermentation [57], but in the experiment, the pretreated corn cob was firstly washed with tap water until neutral pH value. Su et al. achieved a final ethanol concentration of 60.5 g/L at the total substrate loading of 25% (*w*/*w*) using alkaline-pretreated corncob and the fed-batch SSCF process [58], but the pretreated corn cob was also firstly washed with tap water until neutral pH value. This washing before SSCF not only increased the process steps, but also caused sugar loss and water consumption. Compared to the results, we directly produced high concentration ethanol using CCR as substrate without undergoing detoxification treatment, which simplified the process and reduced costs.

## 3. Materials and Methods

### 3.1. Materials and Strains

The pretreated corn stover (ACSE-CS) by acid catalyzed steam explosion (0.1% sulfuric acid, 5 min at 1.7 MPa), and corn cob residues (CCR) obtained through extracting xylose from corn cob with dilute sulfuric acid of 1% (*v*/*v*) under 120 °C for 2 h, followed by lignin removal with ammonium sulfite under the conditions of 12% of ammonium sulfite dosages (on oven dry weight of biomass), 160 °C for 20 min, were provided by Dahe Ecological Technology Co., Ltd. (Liaocheng, China).

The liquid chromatography standards including glucose, xylose, and cellobiose, were purchased from Sigma-Aldrich Co. LLC (St. Louis, MO, USA). Reagent-grade sulfuric acid (98%), anhydrous sodium acetate, sodium hydroxide, and ethanol were purchased from Sinopharm Chemical Reagent Co., Ltd. (Shanghai, China).

The commercial cellulase powder was provided by Sino Biotechnology Co., Ltd., (Baiyin, China). It was diluted 10-fold with a buffer prior to use. The enzymes activities of the commercial cellulase were determined, and they were as follows: filter-paperase activity 159 FPU/g, β-glucosidase 2033 U/g, xylanase 327 U/g, and β-xylosidase 20.2 U/g.

*Saccharomyces cerevisiae* LFB-1 with the ability to co-metabolize glucose and xylose was kindly provided by Professor Yu Shen from Institute of Microbial Technology of Shandong University. The strain was firstly domesticated according to the following method before use: the saccharification solution with inhibitors, which was obtained by enzymatic hydrolysis of ACSE-CS at a solid loading of 30%, was employed as a mother solution. After testing, the saccharification solution contained 91.3 g/L glucose, 44.8 g/L xylose, 0.67 g/L 5-hydroxymethyl furfural, 0.21 g/L furfural, 1.39 g/L formic acid, and 7.37 g/L acetic acid. This solution was diluted with tap water to its concentrations of 25%, 50%, and 75% (*v*/*v*), respectively, to obtain the domestication culture media with different inhibitors concentrations. The activated LFB-1 strain was consecutively domesticated and transferred from the medium with a low inhibitor concentration to the medium with a high inhibitor concentration, until the concentration of inhibitors in the culture medium reached its maximum. Then, a single colony of the resistant strain was screened by spreading it onto a medium plate with the corresponding inhibitors concentration, and designated it as strain A. LFB-1, which was used for SSCF in this study.

### 3.2. Analysis of Chemical Compositions of Lignocellulosic Materials

The chemical compositions of lignocellulosic materials including the contents of cellulose, hemicellulose, and lignin were determined according to the National Renewable Energy Laboratory (NREL) method NREL/TP-510-42618 [59]. Briefly, lignocellulosic biomass was two-step hydrolyzed with 72% and 4% sulfuric acid, respectively, and then was filtered with filtering crucible. The solid residue was regarded as acid-insoluble lignin. The collected filtrate was further filtered using a 0.22 µm Millipore Syringe Filters (Jinteng, China), and then used to analyze the glucose and xylose contents by HPLC (Shimadzu, Japan) equipped with Shimadzu’s 20A refractive index detector, which were used for calculating the contents of glucan and xylan. An Aminex HPX-87P column (Bio-Rad, Richmond, CA, USA) was used to separate the compounds at 85 °C. The mobile phase was ultrapure distilled water with a 0.5 mL/min flow rate.

### 3.3. Fed-Batch Enzymatic Hydrolysis

For fed-batch enzymatic hydrolysis of lignocellulose, the ACSE-CS or CCR, which almost do not contain monosaccharides, was thoroughly mixed with cellulase in a 50 mL flask and incubated at 48 °C in a thermostatic air bath shaker operating at 150 rpm for 72 h. Following the experimental design, substrate or substrate and the commercial cellulase were fed in batches after liquefaction until the final target solid loadings was achieved. For ACSE-CS, the final solid loading was 30% (*w*/*v*), and cellulase dosage was 10 FPU/g DM. For CCR, it was 27% (*w*/*v*) and 8 FPU/g DM, respectively. Timed samples were collected at 24 h, 48 h, and 72 h of enzymatic hydrolysis, and then centrifuged at 12,000 rpm for 10 min to obtain the supernatant for determining different sugar concentrations.

For the enzymatic hydrolysis with addition of Tween 80, the Tween 80 was added all at once into the reaction system at 0 h.

The conversions of glucan and xylan were calculated by Formula (1) and Formula (2) respectively:

Glucan conversion (%) = M1/G × 0.9 × 100%(1)

Xylan conversion (%) = M2/X × 0.88 × 100%(2)

where M1 and M2 represent the amounts of glucose and xylose released from hydrolysis, respectively (mg); G and X denote the theoretical content of glucan and xylan in the substrate, respectively (mg).

### 3.4. Fed-Batch SSCF

Pre-saccharification of pretreated biomass was firstly conducted for determining the initial solid concentration in reaction system. The substrate was thoroughly mixed with diluted cellulase solution in a 50 mL triangular bottle, which was sealed with a rubber plug with an injection needle, and pre-saccharification was conducted at a constant temperature of 48 °C on a shaking table set to a speed of 150 rpm for a duration of 3 h.

After pre-saccharification, the reaction system was cooled to 35 °C, and the *Saccharomyces cerevisiae* A. LFB-1 activated in YPD medium containing 20 g/L glucose, 20 g/L peptone, and 10 g/L yeast extract for 24 h in a thermostat air bath shaker at 35 °C and 200 rpm, was inoculated into the fermentation system with an inoculation volume of 1% (*v*/*v*). The OD_600_ value of the inoculum was 2.0. During this fermentation, a specific ratio of cellulosic substrates was fed in batches into the reaction system according to experiment designs, which was mainly based on the liquefaction situation of the added materials. That is to say, further feeding was initiated after the previously added substrates were completely liquefied, until the target substrate concentration was reached. Fermentation was carried out in a 50 mL flask and incubated for 144 h in a thermostat air bath shaker at pH 4.8, 35 °C, and 200 rpm. Sampling was conducted at regular intervals during SSCF, and the samples were centrifuged at 12,000 rpm for 10 min, and the resulting supernatant was boiled for an additional 10 min to denature the enzyme, and then centrifuged at 12,000 rpm for 10 min and filtered using a 0.22 μm filter membrane. The concentrations of ethanol and sugars in the supernatant were subsequently determined using HPLC.

The HPLC (LC-20AT, RID-20A, Shimadzu, Kyoto, Japan) with HPX-87H columns (Bio-Rad, Hercules, CA, USA) and a differential refractive index detector was used for determining different sugar concentrations in hydrolysates from enzymatic hydrolysis and the contents of residual sugars and ethanol in fermentation broths. The mobile phase was 5 mM H_2_SO_4_ with a flow rate of 0.5 mL/min. The column temperature was maintained at 60 °C.

All experiments were conducted in triplicates, and the average values are reported in the paper.

### 3.5. Analytical Methods

The enzymes activities were determined according to the methods presented by Gao et al. [60], in which the filter-paperase (FPase) activity was measured using a Whatman No.1 filter paper (50 mg) as substrate at 50 °C for 1 h in 2 mL reaction system (1.5 mL buffer + 0.5 mL diluted enzyme solution). The xylanase activity was measured using xylan of 1% (*w*/*v*) as substrate at 50 °C for 30 min. The reduced sugars released were analyzed using the DNS assay. For the activities of β-glucosidase (*p*NPGase) and β-xylosidase (*p*NPXase), using 1 mg/mL (*w*/*v*) of *p*-nitropheny-β-D-glucoside and *p*-nitrophenyl-β-D-xylopyranoside as substrates, respectively, the enzyme solution of 100 μL was mixed with the substrate of 50 μL, respectively, and the reaction was conducted at 50 °C for 30 min, and then terminated by adding 150 μL of 10% Na_2_CO_3_. After it, the OD value of the solution at 420 nm was measured.

All reactions were performed in 50 mM NaAc-HAc buffer of pH 4.8.

One unit (U) of enzyme activity was defined as the amount of enzyme that liberates 1 μmol of reducing sugars (for FPase and xylanase) equivalent or *p*-nitrophenol (for *p*NPGase and *p*NPXase) per minute under the assay conditions.

## 4. Conclusions

By optimizing fed-batch strategies, the problem of the difficulty in enzymatic hydrolysis of cellulosic substrate due to non-liquefaction under high solid loading was overcome, and the high-efficient enzymatic hydrolysis of ACSE-CS and CCR was achieved at the high solid loading of 30% and 27%, respectively, producing the sugar solutions with total fermentable sugar concentrations of 161.2 g/L and 205 g/L, respectively, which are higher than that reported in the literature. Also, using the fed-batch strategy combined with the SSCF process, the ethanol concentrations of 46.1 g/L and 72.8 g/L were obtained using ACSE-CS and CCR as feedstocks under the solid loadings of 35% and 29%, respectively, without undergoing detoxification and washing. This study, especially the production of high concentration fermentable sugar solution, is very valuable for establishing the “sugar platform” based on lignocellulosic biomass, and for promoting efficient bioconversion of lignocellulosic biomass into liquid fuels and bulk chemicals. Further work will be conducted to validate the effectiveness of the processes on an expanded experimental scale for promoting its industrial application.

## Figures and Tables

**Table 1:** Chemical compositions of two lignocellulosic materials (%).

Materials	Glucan	Xylan	Lignin
ACSE-CS	37.8 ± 0.7	18.6 ± 0.3	14.7 ± 0.3
CCR	75.1 ± 0.1	16.7 ± 4.6	8.67 ± 0.05

**Table 2:** Solid loadings and enzyme dosages in reaction system for different feeding strategies.

Feeding Strategies	Hydrolysis Time (h)	Solid Loading (%)	Enzyme Dosage (FPU)
Strategy 1	0–8	10	20
	8–16	17	34
	16–24	24	48
	24–72	30	60
Strategy 2	0–3	15	30
	3–6	23	46
	6–72	30	60
Strategy 3	0–3	15	60
	3–6	23	60
	6–72	30	60

Note: The total dosage of cellulase was 10 FPU/g DM, the enzymatic hydrolysis was performed at 48 °C, pH 4.8, 150 rpm, and the reaction system was 20 mL in 50 mL flask.

## Data Availability

The datasets during the current study are available from the corresponding author on reasonable request.
